# Jefferson Fracture and the Classification System for Atlas Fractures, A Case Report

**DOI:** 10.21980/J88P9C

**Published:** 2021-04-19

**Authors:** Miguel Angel Martinez-Romo, Christopher Eric McCoy

**Affiliations:** * University of California, Irvine, Department of Emergency Medicine, Orange, CA

## Abstract

The Jefferson fracture classification system describes fractures of the atlas (first cervical vertebra or C1). Jefferson fractures with potential tears in the transverse ligament can cause cervical spine instability and can result in neurologic injury if not appropriately diagnosed and managed. We present the case of a 54-year-old man who fell head first with cervical spine tenderness and upper extremity paresthesias. The patient's Jefferson fracture was diagnosed via computed tomography. The patient was then treated non-operatively for his Jefferson fracture, and he had an unremarkable hospitalization. Emergency physicians should obtain surgical consultation and consider the possibility of ligamentous injury in patients suffering injury to the cervical spine.

**Topics:**

Trauma, orthopedics, neurosurgery, cervical fracture, Jefferson fracture.

**Figure f1-jetem-6-2-v16:**
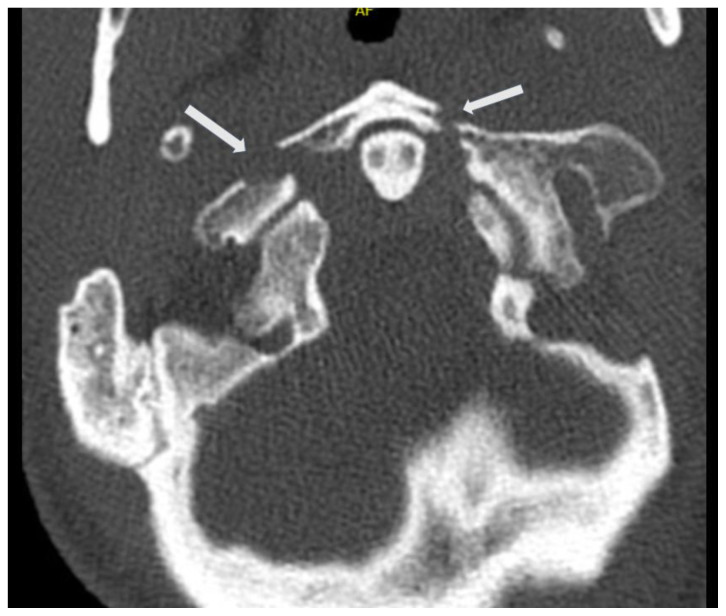


**Figure f2-jetem-6-2-v16:**
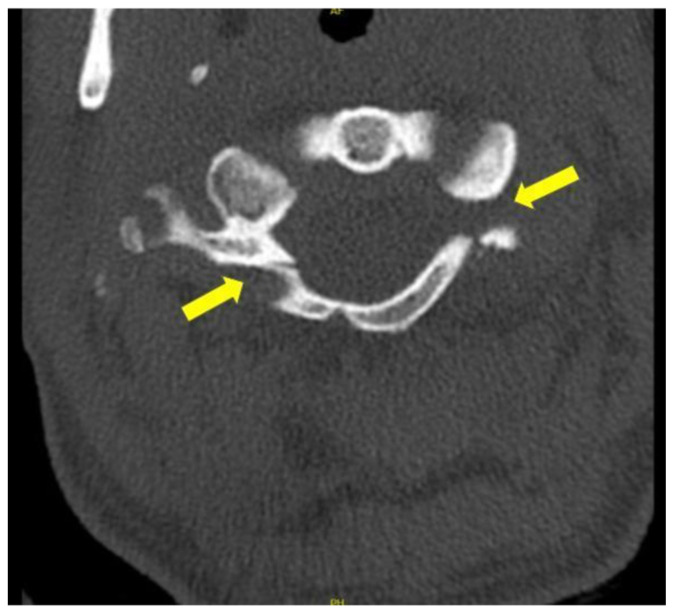


**Figure f3-jetem-6-2-v16:**
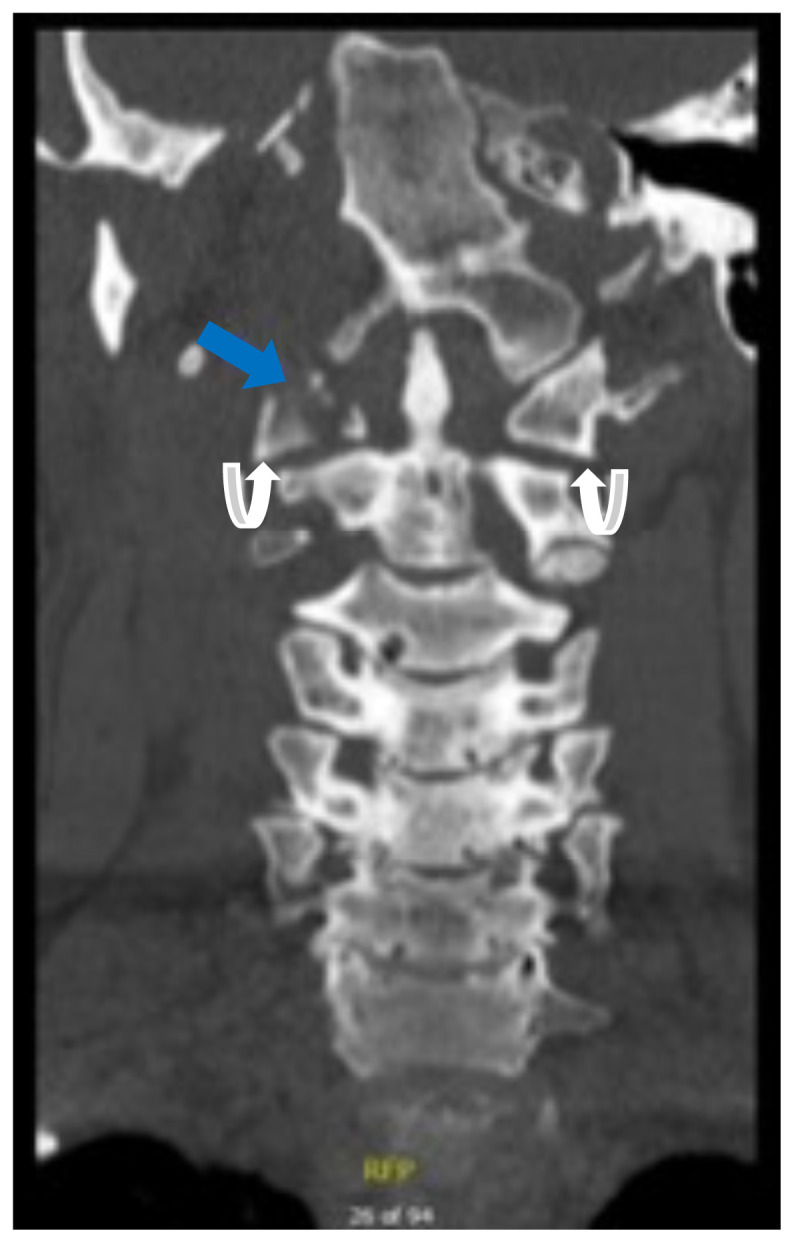


## Brief introduction

[Fig f1-jetem-6-2-v16][Fig f2-jetem-6-2-v16][Fig f3-jetem-6-2-v16]Jefferson fractures occur when a vertical compression force is transmitted through the occipital condyles to the lateral masses of the atlas.^1^ This potentially unstable cervical spine fracture can result in neurologic or vascular injury if not appropriately diagnosed and adequately managed. This case report will present an illustrative case of a patient suffering a Jefferson fracture, present the classification systems for its characterization, and introduce the potential treatment options available for this condition.

## Presenting concerns and clinical findings

A 54-year-old-male presented to a level I trauma center after falling 8 feet from an attic through a ceiling, headfirst. He complained of head, neck, and back pain, along with tingling in his upper extremities. Initial vitals were temperature 36.8 °C, blood pressure 145/91 mmHg, heart rate 70 beats/min, respirations 16/minute, and oxygen saturation of 100% on room air. Physical exam revealed a laceration to the right temporal area, along with cervical, thoracic and lumbar midline tenderness. He had a Glasgow Coma Scale (GCS) score of 15, intact cranial nerves II–XII, five out of five strength in all extremities, and had intact sensation to fine touch throughout his extremities.

## Significant findings

Computed tomography (CT) revealed a burst fracture (Jefferson) of the anterior arch (white arrows) and of the posterior arch (yellow arrows) of the first cervical vertebrae (C1). There was also a fracture of the right lateral mass (blue arrow) of C1 with mild lateral subluxation of the lateral masses (curved arrows).

## Patient course

Inpatient magnetic resonance imaging (MRI) did not reveal an associated ligamentous injury. The patient's Jefferson fracture was treated non-operatively with a hard cervical collar. He had an unremarkable inpatient course without any neurologic deficits or sequelae, and followed-up as an outpatient.

## Discussion

The Jefferson fracture is a fracture of the atlas (C1) that occurs when a vertical compression force is transmitted through the occipital condyles to the lateral masses of the atlas.^1^ Fractures of the atlas with injury to the transverse atlantal ligament can result in cervical spine instability with neurologic injury if not appropriately diagnosed and managed. Neurologic injuries due to spinal cord damage from C1 fractures are rare.^1^ Post-traumatic injury to the lower four cranial nerves (IX–XII) has also been described in case reports (Collet-Sicard Syndrome).^1^,^7^ Vertebral artery injury can also occur and lead to cerebrovascular accidents. 2,8 Prognosis of atlas fractures is in large part dependent on spinal stability, which is supported by the classification of injury.

Patients presenting with a traumatic fracture of C1 may present with upper cervical spine pain, muscle tenderness, muscle spasm, and decreased range of motion of the neck.^1^ On rare occasion they may present with torticollis, cock-robin deformity, or neurologic deficits.^1^

The initial imaging modality of choice for suspected cervical spine fractures in the emergency department is CT rather than radiographs, owing to its superior sensitivity for detecting cervical spine fractures (98.5% vs 43% respectively).^3^,^4^ In cases where ligamentous injury or other soft tissue injury is suspected, further imaging with MRI may be warranted because cervical spine stability is in large part dependent on the integrity of the transverse atlantal ligament.^1^ Nevertheless, the initial CT scan of the cervical spine should provide the emergency physician with sufficient information to appropriately classify the cervical spine injury.

There are several classification systems for atlas fractures available, the 3 most commonly used being the Jefferson classification, followed by the Landells and the Gehweiler classifications.^2^ Knowledge of the different fracture types with their associated prognosis will allow the emergency physician to appropriately manage patients presenting with these injuries. The Jefferson classification system is commonly used in the United States, while the Gehweiler is used widely in Europe.^1^,^2^ The classic 4-part burst fracture seen in our patient is a Type II Jefferson Fracture (Type III Gehweiler).^1^ The patient also had an injury to the lateral mass, consistent with a Jefferson type III and a Gehweiler type IV fracture. It is worth noting that some variability exists in the literature with regard to the numbering of the fractures in the Jefferson classification system (with some texts giving a specific classification for an anterior or posterior arch fracture and some texts stating that either fracture can be classified as type I, as in this article).

Knowing the types of fractures presented in the classification systems is important for the emergency physician because different fracture patterns can have a different prognosis. This knowledge will allow for the appropriate management and disposition for patients found to have an atlas fracture. For example, Type 1 Jefferson Fractures (which correspond to Type I and II Gehweiler) are usually stable and can be treated with a hard collar. 1,2 Type II and III Jefferson Fractures (Type III and IV Gehweiler) can be associated with transverse ligament injury and/or severe dislocation and thus instability.^1^,^2^ Consultation with a spine surgeon will allow for the appropriate management and disposition for patients with these types of cervical spine injuries.

Stable fractures may be treated with a cervical collar and unstable fractures with osteosynthesis of the atlas or halo-traction, although the choice for conservative versus surgical treatment is controversial.^1^,^6^,^9^ Since the integrity of the transverse ligament is not definitively known, even after CT, the emergency physician should assume the possibility of ligamentous injury and take spinal precautions. Orthopedic or neurosurgical consultation is warranted because the integrity of the transverse ligament is likely not to be known in the emergency department, and for the evaluation of conservative versus surgical treatment.^6^,^9^[Table t1-jetem-6-2-v16]

**Table t1-jetem-6-2-v16:** Jefferson Classification:^1^,^2^

C1 (Atlas) Fracture	Jefferson Classification
Isolated anterior OR posterior arch fracture	Type I
Fracture of the anterior AND posterior arch	Type II
Fracture of lateral mass with or without fracture of the posterior arch	Type III

**Table t2-jetem-6-2-v16:** Gehweiler Classification: 1,2

C1 (Atlas) Fracture	Gehweiler Classification
Isolated fracture of the anterior arch	Type I
Isolated fracture of the posterior arch	Type II
Combined fracture of the anterior and posterior arch	Type III
Fracture of the lateral mass	Type IV
Fracture of the transverse process	Type V

[Table t2-jetem-6-2-v16]

## Supplementary Information












