# Supplementary figures and images for: Jefferson Fracture and the Classification System for Atlas Fractures, A Case Report
Source: J Educ Teach Emerg Med. 2021 Apr 19;6(2):V16–9. doi: 10.21980/J88P9C (PMC10332790; doi:10.21980/J88P9C)

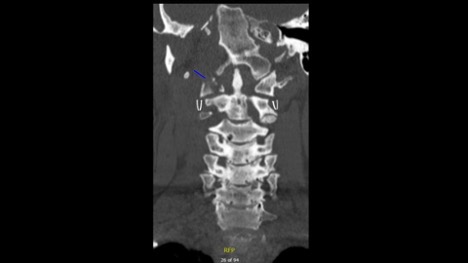

Supplement: Supplementary file 1 [file jetem-6-2-v16-supp1.jpg]

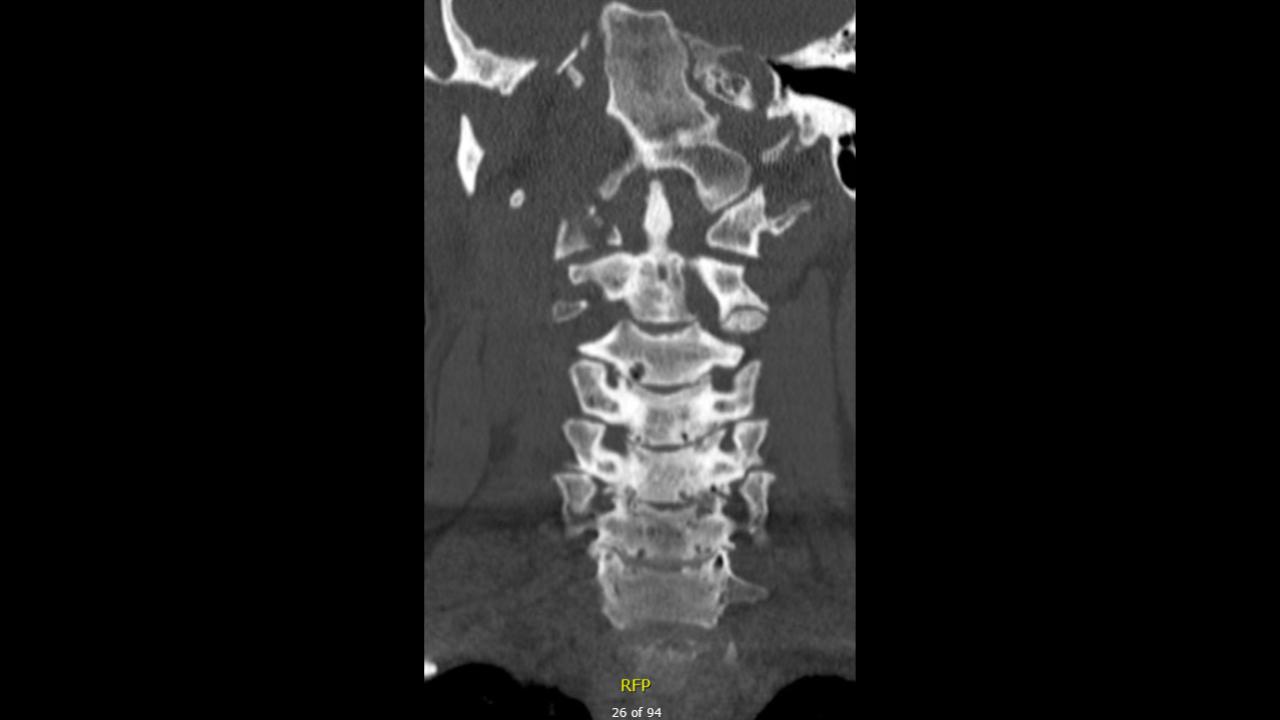

Supplement: Supplementary file 2 [file jetem-6-2-v16-supp2.jpg]

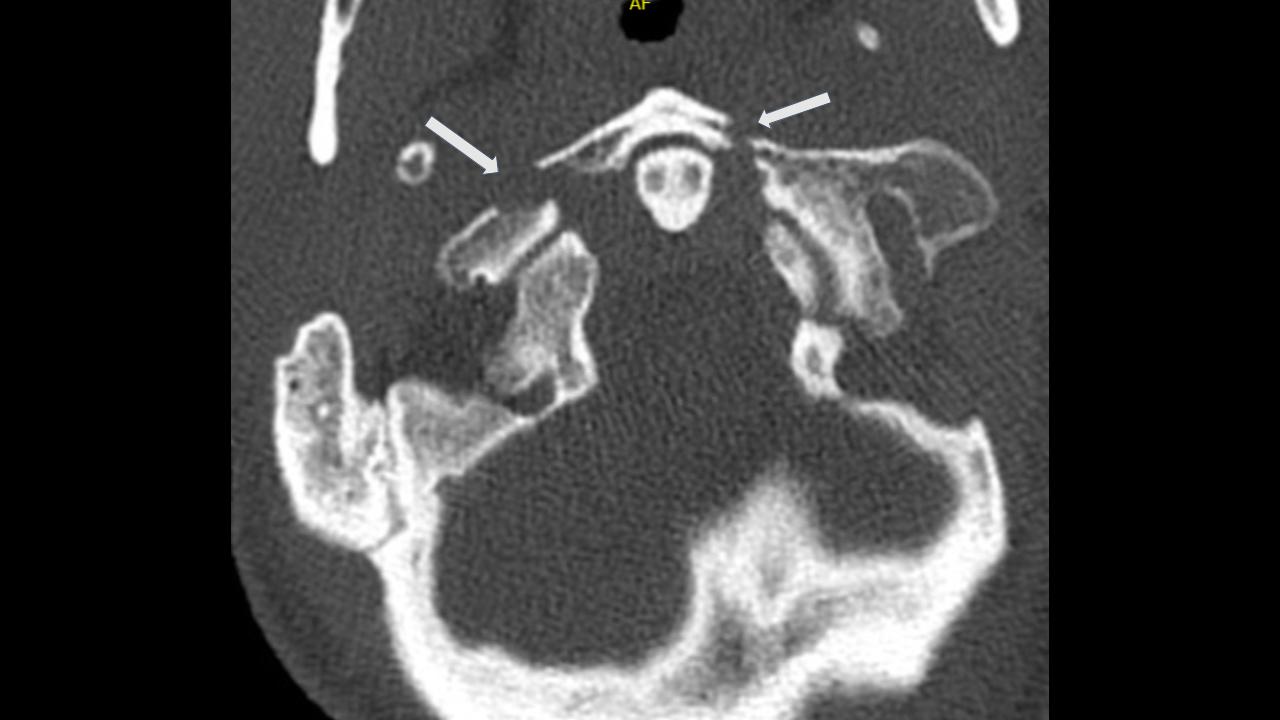

Supplement: Supplementary file 3 [file jetem-6-2-v16-supp3.jpg]

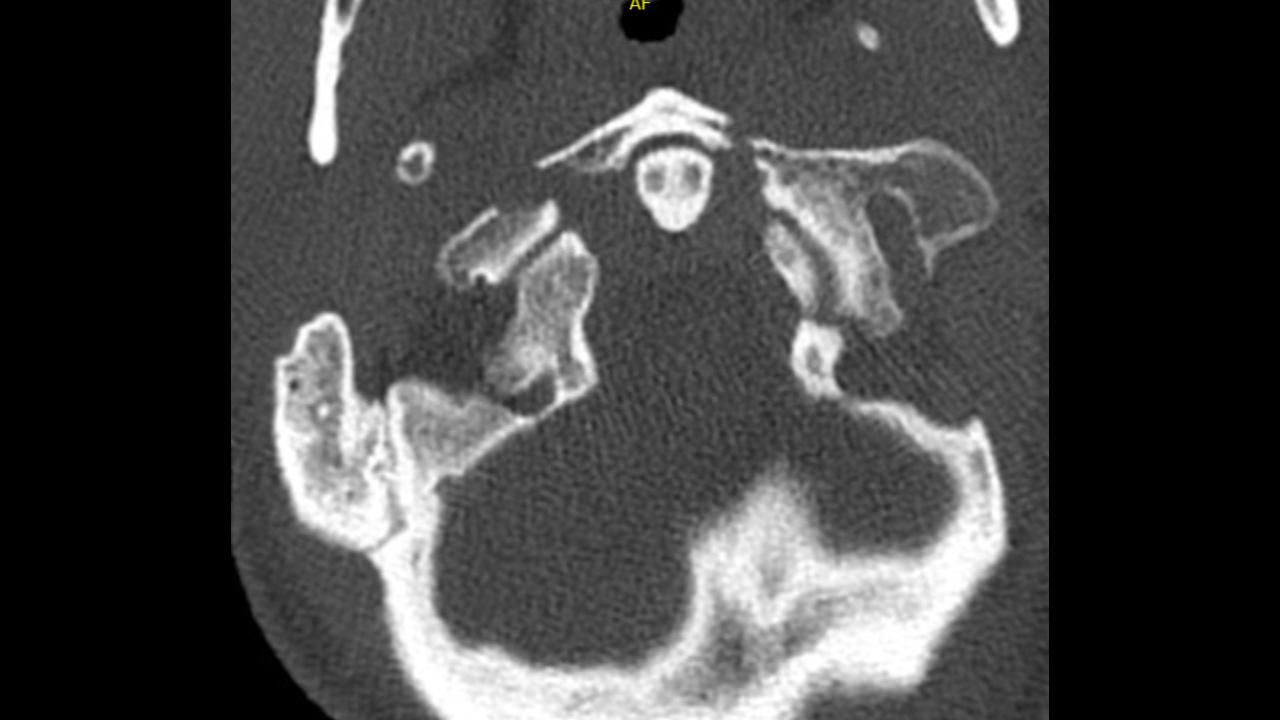

Supplement: Supplementary file 4 [file jetem-6-2-v16-supp4.jpg]

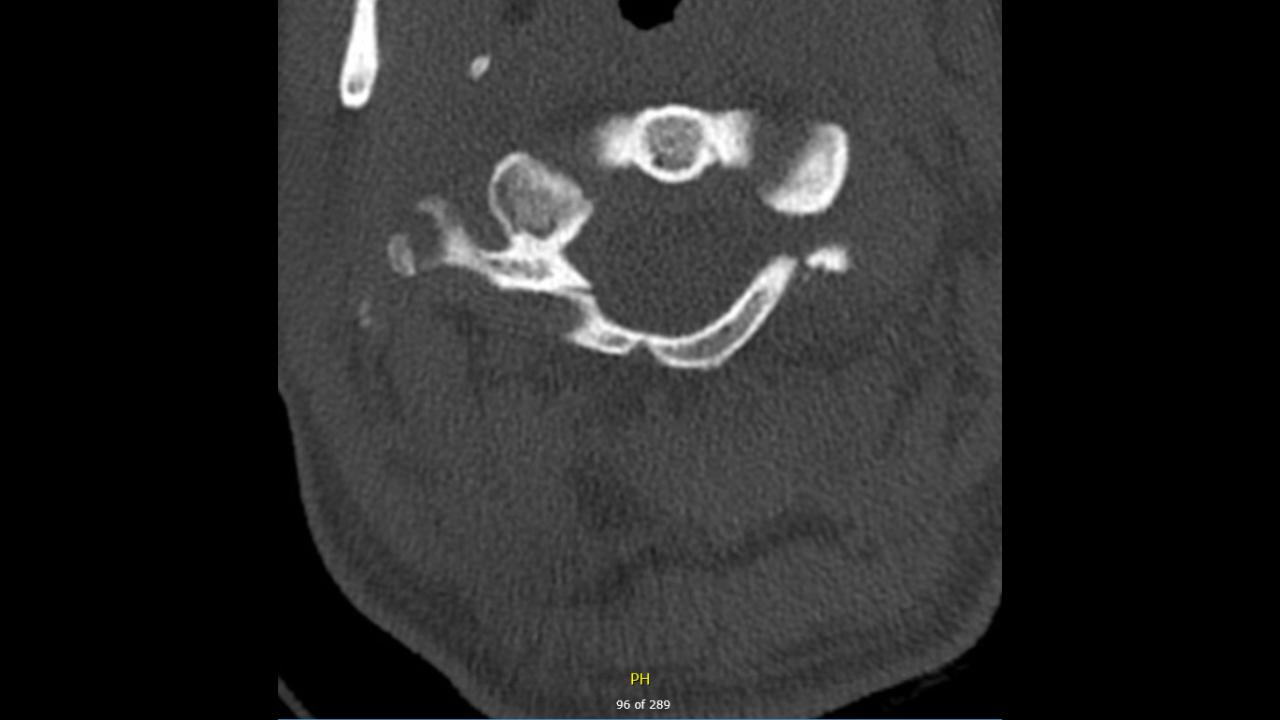

Supplement: Supplementary file 5 [file jetem-6-2-v16-supp5.jpg]

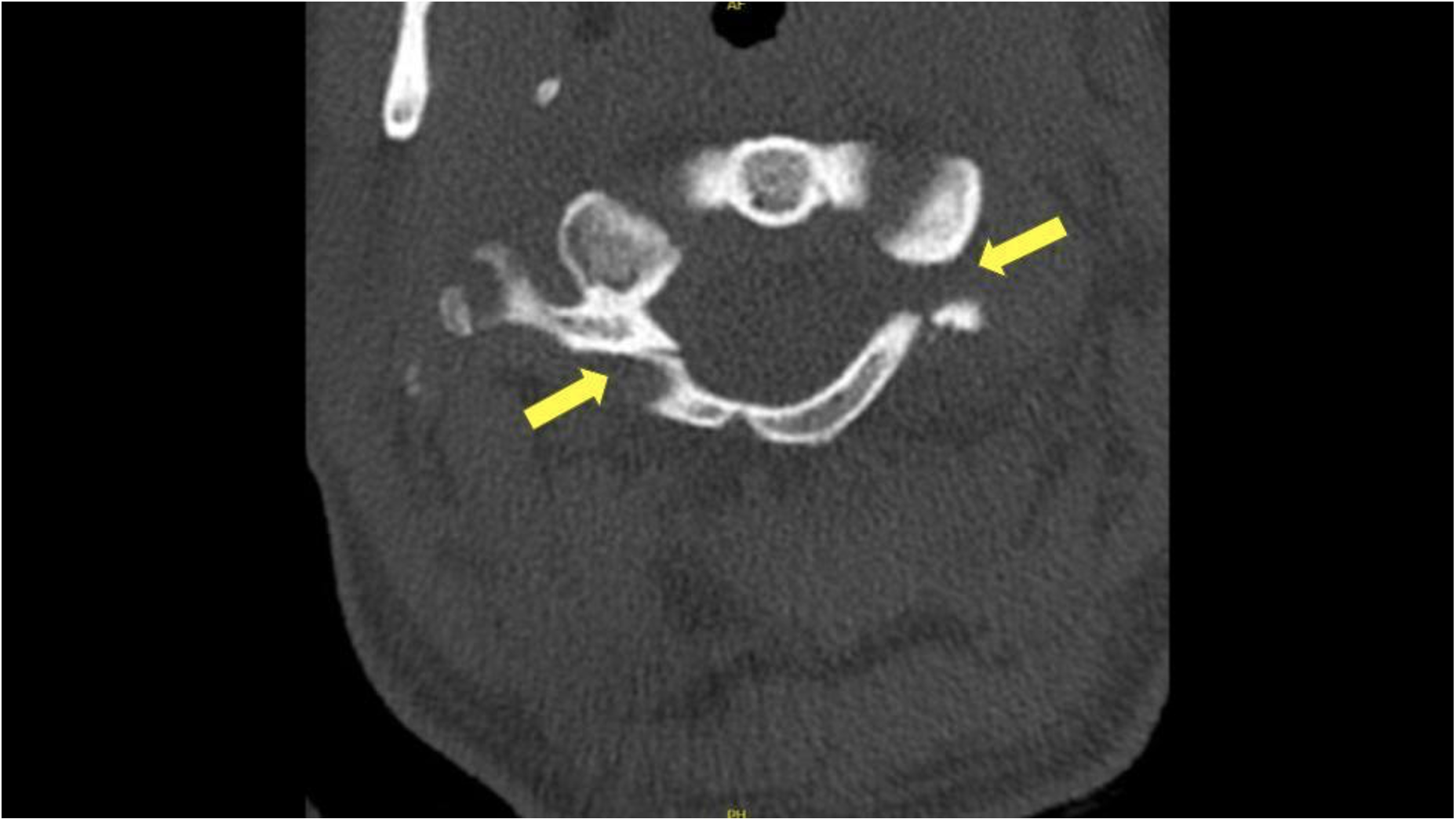

Supplement: Supplementary file 6 [file jetem-6-2-v16-supp6.png]
